# Living to our full potential: Reassessing global sex inequalities in life expectancy

**DOI:** 10.1371/journal.pmed.1004987

**Published:** 2026-03-17

**Authors:** Ann M. Weber, Gary L. Darmstadt

**Affiliations:** 1 Department of Epidemiology, Biostatistics and Environmental Health, School of Public Health, University of Nevada, Reno, Nevada, United States of America; 2 Department of Pediatrics, Stanford University School of Medicine, Stanford, California, United States of America

## Abstract

In this Perspective article, Ann Weber and Gary Darmstadt discuss how benchmarking life expectancy against what is achievable reveals how sex disadvantage shifts by age, place, and time, and reframes inequality as unrealized potential due to social and structural constraints rather than differences in biology.

It is widely understood that, on average, females suffer more illness-related disability than males throughout the life course [1]. For example, in 2021, three nonfatal diseases (lower back pain, depressive disorders, and headache disorders), which are reported more commonly by females than males, accounted for the largest burden of years lived with disability for all ages and sexes globally [1]. Conversely, females on average tend to live 3–5 years longer [2]. Despite extensive evidence, controversy surrounds the nature of the “problem” with differences in health by sex (here defined as biological and physiological factors associated with being male or female), with consequently little action in achieving health equity [3]. The problem is further mired by recent erroneous rejections of gender (the social construct that influences the roles, behaviors, and identities of all people) as a separate construct from sex [4] despite evidence that for most health conditions, sex cannot explain differences in the absence of considerations of gender [5,6].

A recent study in *PLOS Medicine* [7] aimed to bring clarity to the problem of sex-based differences in health by developing an objective method of evaluating differences in life expectancy (LE) across age groups and countries. The authors use an innovative approach that “sets the frontier” or limit for what is currently possible in LE based on countries in the top 5th percentile of LE, disaggregated by age and sex. The ratio of female to male LE by age group among the frontier countries becomes a reference point to evaluate disparities in sex ratios of LE for each age group in a country. The distance of a country’s LE by sex from this achievable ratio thus identifies the extent of disadvantage in LE by sex and age. Having objective, age-specific sex ratios as reference points differentiates the authors’ approach from others that reference a sex ratio of 1 at all ages (except at birth [8]).

This study makes an important contribution in effectively challenging the perception of a female survival advantage over males globally [9]; while males generally are more disadvantaged in LE than females, their method shows that disadvantage shifts from male to female in many countries and age groups, and over time. For example, in Central and Eastern Europe, males are disadvantaged across most ages, except in Albania, where females are consistently disadvantaged. Meanwhile, males are more consistently disadvantaged across countries at young ages, while female disadvantage often emerges with age. For example, in Mexico, males are disadvantaged from birth to age 35, but females are disadvantaged at age 70. Sex differences in LE have also shifted over time: between 2000 and 2019, male disadvantage at birth shifted to female or no disadvantage in 32% of countries relative to the frontier, although the reasons for the shift vary relative to the starting context, including improvements in female LE (e.g., in Ethiopia), improvements in male LE in frontier countries (e.g., in China), and stagnation or decline in male LE (e.g., in Syria). These results therefore demonstrate that there is no universal rule for the direction of sex disadvantage in LE (Fig 1).

**Fig 1:**
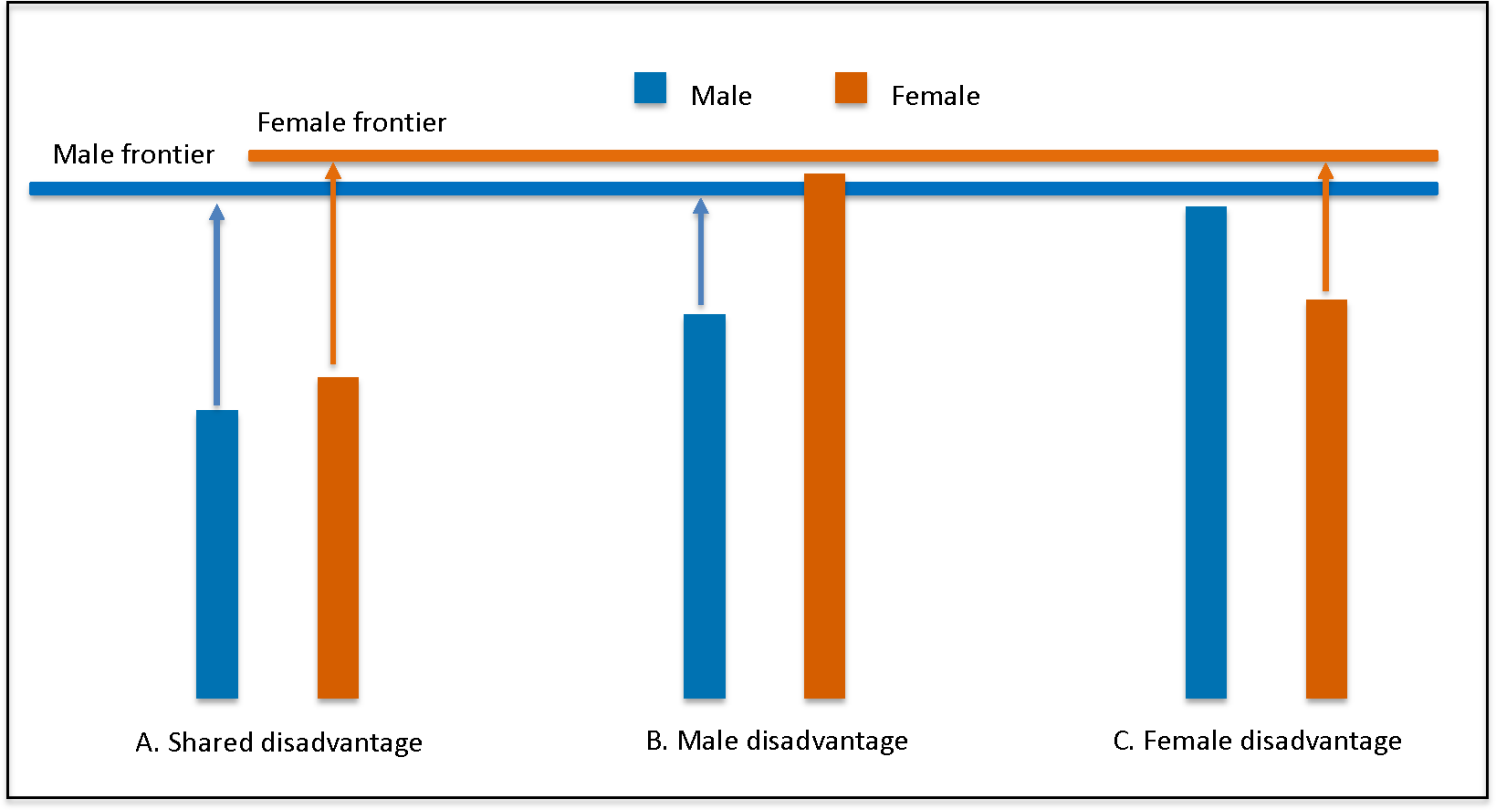
Conceptual illustration of observed vs. frontier life expectancy (LE) by sex under three scenarios. Scenarios A, B, and C could represent three different countries or three different ages across the life span*. Scenario A: observed LE for both sexes falls below their potential. Scenario B: males fall below their potential. Scenario C: females fall below their potential. The vertical bars represent observed life expectancy (LE) for males (blue) and females (orange). The horizontal lines represent frontier LE for males (blue) and females (orange). The arrows represent the shortfall in LE between what is observed and the frontier of what is possible. *AI assistance was used to support figure conceptualization and drafting. No data analysis or interpretation was performed by the tool.

A key insight that emerges from this analysis is that in most countries and at most ages, both sexes are constrained from achieving what is *possible* (see Fig 1). Important questions that then arise are why, and what can be done about it? Setting LE frontiers for how long people *can live* parallels setting standards for how children *can grow* if given the opportunity to reach their full potential, without restrictions on their growth. Child growth standards developed by the World Health Organization (WHO) in the early 2000’s [10], and fetal growth standards developed more recently [11], have changed how we measure, interpret and act on maternal and child nutrition and health worldwide, influencing our scientific thinking, programmatic planning, and policy decision-making [12]. Could setting a frontier for LE by sex similarly change how we interpret and act on the failure for populations to achieve their potential?

Frontier LE could be interpreted as the biological limit of what is achievable given the most favorable healthcare, nutrition, social, cultural, and environmental conditions that exist in our world today. Then, the gap between observed LE and frontier LE at any age for either sex in a country is not just about biology. Instead, the gap primarily represents a deficit in what is achievable due to a mix of other constraints on LE, some of which may be gendered or intersect with sex, but others are not.

Fig 1 shows a conceptual illustration of the shortfall in LE between what is observed and possible by sex under three hypothetical scenarios. The scenarios could reflect LE in different countries for a single age or in one country for different ages (or other demographic variables such as race/ethnicity or wealth). In scenario A, the observed LE for both sexes falls below their potential, as is in many sub-Saharan countries [7]. The sex difference is small in comparison to the shortfall, suggesting that shared constraints affect both sexes (it’s not just about sex or gender). In this scenario, an emphasis on sex disparities could fail to recognize and address population-level constraints that affect everyone though a variety of mechanisms that may or may not act similarly in males and females, but nevertheless act together to reduce LE for all. In scenario B, the observed LE for males falls short, but females are closer to achieving their potential (as seen in Belarus and Georgia), suggesting that constraints that disproportionately affect males should take priority; these constraints may act through sex-based factors and/or via gendered determinants of health (e.g., norms for masculine behaviors). In contrast, scenario C suggests that female-specific constraints need attention, for example, for older women in northern Europe [7].

The authors write that their approach was developed to “analyze and interpret sex inequalities in health outcomes,” and provide a broad framework for approaching local, regional, and global data through a gender lens. As such, the findings offer important insights that demand deeper investigation into what is needed to allow everyone to live to their full potential. However, the findings don’t tell us what to do or for whom; they cannot explain the complex and intertwined mechanisms shaping survival for a given sex, age group, or country. Critically, country-level estimates may overlook within-country inequities (e.g., rural female disadvantage offset by urban female advantage) that should be prioritized. Additional multisectoral quantitative and qualitative data are needed to define more specific contributions of various structural, economic, environmental, social, and cultural determinants of health and how they intersect with sex, gender, and age in creating the shortfall from the frontier.

Another limitation is that a focus on LE informs relative sex disadvantage of *length* of life lived, but does not capture disadvantages in *quality* of health during those years lived. Would more people benefit if we examined sex differences in a measure that reflects years lived in health, free of disability, such as Healthy Life Expectancy (HALE)? We posit that in settings where LE falls far short of what is possible, HALE and LE findings are likely to be aligned; a life curtailed prematurely contributes fewer years lived with potential disability, especially if death occurs during childhood or early adulthood. In contrast, when LE is near the frontier of possibility, examining quality of life may generate more actionable information. However, it is important to keep in mind that disability weights used to estimate HALE are “universal” (i.e., fixed by age, sex, and context) and may underrepresent true burden and related disparities in certain settings and for certain conditions [13,14]. Therefore, examining HALEs or other quality of life measures (e.g., quality-adjusted life years), while informative, would not alleviate the need for in-depth investigation to understand the source of differences, as noted above.

While sex and gender analyses can seldom stand in isolation in informing public health actions, their consideration is a critical component of a broader analysis of a complex, interacting set of factors across individual biology and physiology as well as contextual determinants of health. The method presented by Chang and colleagues [7] effectively debunks broad-stroke assumptions about female or male disadvantage—there are no universal winners or losers by sex. They also provide an important catalyst and input as we commit to the unfinished global agenda of ensuring that all people, individually and collectively, live to their full potential.

## Abbreviations

HALE: Healthy Life Expectancy
LE: life expectancy
WHO: World Health Organization.

## References

[1] GBD 2021 Diseases and Injuries Collaborators. Global incidence, prevalence, years lived with disability (YLDs), disability-adjusted life-years (DALYs), and healthy life expectancy (HALE) for 371 diseases and injuries in 204 countries and territories and 811 subnational locations, 1990-2021: a systematic analysis for the Global Burden of Disease Study 2021. Lancet. 2024;403(10440):2133–61. doi: 10.1016/S0140-6736(24)00757-8 38642570 PMC11122111

[2] GBD 2019 Demographics Collaborators. Global age-sex-specific fertility, mortality, healthy life expectancy (HALE), and population estimates in 204 countries and territories, 1950-2019: a comprehensive demographic analysis for the Global Burden of Disease Study 2019. Lancet. 2020;396(10258):1160–203. doi: 10.1016/S0140-6736(20)30977-6 33069325 PMC7566045

[3] Hawkes S, Sy EA, Barker G, Baum FE, Buse K, Chang AY, et al. Achieving gender justice for global health equity: the Lancet Commission on gender and global health. Lancet. 2025;405(10487):1373–438. doi: 10.1016/S0140-6736(25)00488-X 40209736

[4] The White House. Defending women from gender ideology extremism and restoring biological truth to the federal government [Cited 16 January 2026]. Available from: https://www.whitehouse.gov/presidential-actions/2025/01/defending-women-from-gender-ideology-extremism-and-restoring-biological-truth-to-the-federal-government/

[5] Weber AM, Cislaghi B, Meausoone V, Abdalla S, Mejía-Guevara I, Loftus P, et al. Gender norms and health: insights from global survey data. Lancet. 2019;393(10189):2455–68. doi: 10.1016/S0140-6736(19)30765-2 31155273

[6] Cislaghi B, Weber AM, Gupta GR, Darmstadt GL. Gender equality and global health: intersecting political challenges. J Glob Health. 2020;10(1):010701. doi: 10.7189/jogh.10.010701 32257161 PMC7101083

[7] Chang AY, Johnson EK, Bolongaita S, Buse K, Hawkes SJ, Karlsson O, et al. From sex differences to sex inequalities in life expectancy: a cross-country observational benchmarking analysis. PLoS Med. 2025;22(12):e1004828. doi: 10.1371/journal.pmed.1004828 41379774 PMC12697978

[8] Hesketh T, Xing ZW. Abnormal sex ratios in human populations: causes and consequences. Proc Natl Acad Sci U S A. 2006;103(36):13271–5. doi: 10.1073/pnas.0602203103 16938885 PMC1569153

[9] Cullen MR, Baiocchi M, Eggleston K, Loftus P, Fuchs V. The weaker sex? Vulnerable men and women’s resilience to socio-economic disadvantage. SSM Popul Health. 2016;2:512–24. doi: 10.1016/j.ssmph.2016.06.006 29349167 PMC5757782

[10] de Onis M, Garza C, Onyango AW, Rolland-Cachera M-F, le Comité de nutrition de la Société française de pédiatrie. WHO growth standards for infants and young children. Arch Pediatr. 2009;16(1):47–53. doi: 10.1016/j.arcped.2008.10.010 19036567

[11] Villar J, Cheikh Ismail L, Victora CG, Ohuma EO, Bertino E, Altman DG, et al. International standards for newborn weight, length, and head circumference by gestational age and sex: the Newborn Cross-Sectional Study of the INTERGROWTH-21st Project. Lancet. 2014;384(9946):857–68. doi: 10.1016/S0140-6736(14)60932-6 25209487

[12] World Health Organization. Global targets 2030 [Cited 23 December 2025]. Available from: https://www.who.int/teams/nutrition-and-food-safety/global-targets-2030

[13] Pigeolet M, Franco H, Nussbaum L, Corlew DS, Meara J. Context matters for disability and priority setting for musculoskeletal diseases: revisiting the egalitarian approach to disability weights and disability-adjusted life-years. BMJ Glob Health. 2023;8(6):e012106. doi: 10.1136/bmjgh-2023-012106 37311581 PMC10277058

[14] Salomon JA, Vos T, Hogan DR, Gagnon M, Naghavi M, Mokdad A, et al. Common values in assessing health outcomes from disease and injury: disability weights measurement study for the Global Burden of Disease Study 2010. Lancet. 2012;380(9859):2129–43. doi: 10.1016/S0140-6736(12)61680-8 23245605 PMC10782811

